# Evaluation of the Acute Flacid Paralysis (AFP) Surveillance System in Bikita District Masvingo Province 2010

**DOI:** 10.1186/1756-0500-7-252

**Published:** 2014-04-18

**Authors:** Kufakwanguzvarova W Pomerai, Robert F Mudyiradima, Mfuta Tshimanga, Mary Muchekeza

**Affiliations:** 1 Department of Community Medicine, University of Zimbabwe, P O Box A178, Avondale, Harare, Zimbabwe; 2 Provincial Medical Directorate, Masvingo Province, Zimbabwe

**Keywords:** Acute flacid paralysis, Surveillance, Bikita

## Abstract

**Background:**

AFP is a rare syndrome and serves as a proxy for poliomyelitis. The main objective of AFP surveillance is to detect circulating wild polio virus and provide data for developing effective prevention and control strategies as well planning and decision making. Bikita district failed to detect a case for the past two years.

**Findings:**

A total of 31 health workers from 14 health centres were interviewed. Health worker knowledge on AFP was low in Bikita. The system was acceptable, flexible, and representative but not stable and not sensitive since it missed1 AFP case. The system was not useful to the district since data collected was not locally used in anyway. The cost of running the system was high. The district had no adequate resources to run the system. Reasons for not reporting cases was that the mothers were not bringing children with AFP and ignorance of health workers on syndromes captured under AFP.

**Conclusion:**

Health worker’s knowledge on AFP was low and all interviewed workers needed training surveillance. The system was found to be flexible but unacceptable. Reasons for failure to detect AFP cases could be, no cases reporting to the centres, lack of knowledge on health workers hence failure to recognise symptoms, high staff turnover.

## Introduction

Surveillance is defined as ongoing, systematic collection, analysis, and interpretation of data and the distribution to those who need to know [1]. This means the dissemination of information that results from properly executed surveillance to those who plan public health programmes, to those who develop local, regional, national and international policies, to those who implement intervention and carry out public health action, to the public who need to have information in order to evaluate public health practice, and to those who need the information for personal action for their health and well being [1]. Surveillance whether active or passive is a dynamic process. It is fundamental to public health decision making and subsequent action. Many factors can change and as a dynamic process surveillance often needs adjustments. Evaluation of surveillance is advisable on a cyclic basis and should be done objectively. No single surveillance is perfect but a combination of approaches work best [1].

A major pre requisite for polio free certification by the World Health Organization (WHO) is that the local AFP surveillance system successfully detects one case of non polio acute flaccid paralysis(AFP) per 100 000 children below fifteen years per annum and that no polio cases are occur for three consecutive years [2]. The goal of AFP surveillance is to detect polio virus within the population and communities and provide accurate data for developing appropriate supplementary vaccination and other preventive strategies. AFP surveillance adequacy and quality is evaluated by the following key performance indicators recommended by WHO;

a) Timeliness and completeness of reporting, at least 80% of expected routine weekly AFP surveillance reports should be received on time, this has to include zero reporting where no AFP cases are experienced. Representativeness has to be considered on the distribution of reporting centers in terms of geographical and demographical characteristics of the country or district.

b) Completeness of case investigation, all AFP cases should have a complete clinical and virological investigation and at least 80% AFP cases having two adequate stool specimens (24–48 hours apart) these are collected for enterovirus studies within fourteen days of onset of symptoms.

c) Laboratory performance, all virological studies on AFP cases must be done in a laboratory certified by the Global Poliomyelitis Laboratory Network(GPLN).

d) Sensitivity of surveillance, at least one case of non polio AFP should be detected annually per 100 000 population under the age of fifteen years.

e) Completeness of follow up, at least 80% of AFP cases should have a follow up examination for residual paralysis at 60 days after the onset of paralysis [3].

### AFP surveillance in Zimbabwe

Zimbabwe and other African countries adopted the polio eradication initiative of the WHO at the National Expanded Programme on Immunisation Meeting Held in Windhoek, Namibia 2004. They adopted the following targets in their AFP surveillance as a way towards attaining polio free certification.

• An AFP detection rate of at least two cases for every 100 000 children below the age of fifteen tears. This was done so as to increase the index of suspicion and hence increase chance of detecting AFP towards eradication.

• There should be at least 90% stool adequacy for all samples of suspected AFP cases. These should be sent to the National Virology laboratory. The requirements are two specimens per case. The specimens should be 24 hours apart, and should be collected within 14 days of onset of paralysis. The specimen should be kept at temperatures of between 2 and 8 degrees Celsius and arrive at the laboratory within 72 hours of collection [4].

There was a AFP surveillance improvement in Zimbabwe from 2000 to 2003 with a consistent detection rate of 80% and above. There was also a stool adequacy improvement from 78 to 83% by end of 2003. There was a decline of the indicators from 2004 onwards [5]. In 2011 the Ministry of Health EPI report reported that the country was failing to meet their target since 2004 [5].

### Objectives of the surveillance system

1. To provide information to guide the development of appropriate strategies towards eradication of polio.

2. To identify areas of transmission of Poliovirus.

3. To investigate all cases of AFP and prove absence of wild polio virus in the country.

4. To measure the impact of intervention such as routine immunization and supplementary immunization activities against polio.

Under the syndromic approach to AFP,a case is defined as any person aged 14 years or younger who presents with a history of recent floppy, inability to use limbs irrespective of what the final outcome will be, or any person of any age diagnosed of polio by a medical officer. This excludes cases due trauma or injury. The following criteria is used for diagnosis;

1. Polio virus found in stool specimen of the case or contact.

2. Death, especially 10 days after onset of symptoms and if difficulty in breathing was reported by the deceased.

3. Presence of residual paralysis after 60 days of symptoms and

4. Patient lost to follow up.

There is great need for 60 day follow up because, if it is not done it may result in misdiagnosis of a false positive case to be a positive case. All cases of AFP cases must be notified, correct case investigation forms filled accurately and properly for surveillance purposes and also adequate stool specimen taken for virological tests.

According to World Health Organization (WHO) “Acute flaccid Paralysis (AFP) detection rate of two per 100 000 children under 15 years and stool adequacy rate of at least 80% and above” is required for a good surveillance system. The country is looking forward to be certified polio free by WHO, and failure by Bikita district (<15 yr population 102000) to detect at least 2 cases per year since 2007 is very disturbing, may lead to the country’s failure to be certified polio free and lead to so many undetected cases of polio in the community. It is against this background i intent to evaluate the surveillance system of Bikita

## Materials and methods

A descriptive cross-sectional study was carried out in Bikita district (worst performing district) of Masvingo province. Fourteen of the twenty two clinics randomly selected using the lottery method where each centre was assigned a number and the numbers put in a box, the numbers where then pick after shuffling until 14 clinics were picked and four of the five hospitals were purposively selected. Health workers involved in the system were conveniently sampled (those on duty at the selected centres) and interviewed. A total of 31 health workers who were found on duty were recruited into the study. The District Nursing Officer (DNO), Health Information Clerk (HIC), District Environmental Health Officer(DEHO) and the District Medical Officer (DMO) were interviewed as key informants.

A pre tested interviewer-administered questionnaire was used to elicit information from health workers to determine their knowledge on the operations and usefulness of the surveillance system. Cost of running the surveillance system was done by listing all the resources required to run the surveillance system and then these resources were costed as per the prevailing price(in US Dollars) as reported by purchasing department in Bikita district. A checklist was used to assess the system’s stability resources available for use in the surveillance system. Records of patients 0–14 years who were admitted at the hospital were reviewed to check on how many AFP cases were seen how many were captured by the surveillance system and how many were missed. Epi info was used to calculate proportions and measures medians.

Permission to carry out the study was obtained from the, Medical research Council of Zimbabwe, Health Studies Office, PMD Masvingo and DMO Bikita. Written consent was obtained from all study participants. Confidentiality was assured and maintained throughout the study.

## Findings

Fourteen of the 22 health facilities were randomly selected into the study. Thirty one participants out of a total two hundred were enrolled into the study. Majority (74.2) of participants were females and were nurses. The median years in services was 16(Q_1_ = 14,Q_3_ = 20) and the median age of participants was 36(Q_1_ = 28,Q_3_ = 46) as shown in Table 1 attached. Knowledge on AFP surveillance was low among health workers in Bikita as shown in Table 2. Knowledge on time required to completely investigate a case and when to do follow up was low 32% and 29% respectively.

**Table 1:** Demographic characteristics of health workers in Bikita district 2010

**Variable**	**Category**	**Total**	**%**
Sex	Male	8	26.8
	Female	23	74.2
Years in service	Median years in service = 16 (Q_1_ = 14,Q_3_ = 20)		
Age in years	Median age in years 36 (Q_1_ = 28,Q_3_ = 46)		
			
Occupation	Doctors	1	3.2
	Matron	1	3.2
	Sister in Charge	3	9.7
	General Nurse	11	35.5
	Primary Care Nurse	13	41.9
	Lab tech/Scientist	1	3.2
	Health Inspector	1	3.2

**Table 2:** Knowledge of AFP surveillance by health workers; Bikita district 2010

**Variable**	**Total**	**%**
AFP acronym	29	93.5
Number of forms to be filled	18	58.1
Target population	18	58.1
Where to sent forms	26	83.9
Specimen collected	28	90.3
Number of specimen taken	23	74.2
Times specimen collected	22	71.0
Temperature at which specimen transported	27	87.1
Time within specimen should reach National laboratory	20	64.5
Time required to completely notify and investigate AFP case	10	32.0
When to follow up a case	9	29.0

### Usefulness

Despite the fact that the system did not detect cases in the past two years (2008 & 2009), the DMO, DNO, DEHO and 96% of the health workers reported the system to be useful. Only two participants mentioned that they did not know whether the system was useful or not. Meetings were not held in the district with only three health workers reporting having held meeting though the minutes were not seen, in addition there was no local use of AFP surveillance data. However only 1 centre reported that they use the data to do awareness campaigns.

### Simplicity

Of the 31 health workers we interviewed only two (6.7%) had filled in a AFP case notification form. For those two who filled the AFP notification forms both reported taking less than 20 minutes to fill the forms and that the forms are not difficulty to fill. However 31(100%) felt they need to be trained in filling the AFP surveillance forms.

### Acceptability

Out the 31 health worker we interviewed 30(96.8%) felt that it was their duty to filling AFP notification forms, and 30(96,8%) also felt they are still willing to continue with the duty of filling AFP surveillance forms.

### Stability

Out of the 31 participant in the study only two (6.5%) reported having AFP notification forms at their health centres, none of the participants were trained in AFP/EPI surveillance, 31(100%) reported using personal cell phone to contact the district office. On transportation of specimen 14(45.2%) reported using hospital vehicle the rest use public transport. Only two centres had a functional telephone.

### Flexibility

All the 31(100%) respondents reported that the AFP notifying forms were flexible since they have space for other conditions.

### Representativeness

Majority of the centres were rural health centres with the exception of the two mission hospital. There are no private clinic or hospital in the district.

### Data quality

Since there were no forms that were filled, data quality assessment was not done.

### Timeliness

In 2008 and 2009 the system failed to detect any case so timeliness was not measured.

### Sensitivity

Reviewed records of inpatients for the under 15 years 1 case was found at a mission hospital. Therefore the system was not sensitive.

Only two of the 14 visited clinics had a functional telephone in Bikita district. The total anticipated cost of effectively running the AFP surveillance system in Bikita is US$10384 per year as shown in Table 3 and would be covering the above stated activities. Hyper inflation is no longer anticipated since the country is now using a more stable currency the United States dollar(USD). The cost of sending forms and other items is based on the prevailing charges by public transport in the district since most clinics use public transport for this activity.

**Table 3:** Anticipated cost of operating the AFP surveillance system in Bikita district Masvingo Province 2010

**Activity**	**Calculation unit cost in USD**	**Total cost US dollars**
Printing and stationery	15 for 22 health facilities per month × 12	3960
Repairs and maintenance	1000 telephones and two way radio communication	1000
Sending forms to the next level	6 per RHC/per month × 12	1584
Training and workshop	50×60 ×1 workshop per year	3000
Vehicle maintenance	200×3 times per years	600
Support and supervision	20×3×4	240
**Total cost**		**USD 10384**

All the health workers reported that no AFP cases occurred during the period under review that’s why they never reported the cases. On the other hand 10(32.2%) reported that mothers in the community were not bringing their children with AFP to the clinic, furthermore 19(61%) reported that they were not aware of syndromes under AFP.

## Discussion

The study revealed that knowledge of AFP surveillance system operations were low in Bikita district except on four areas,the AFP acronym (93.5%), type of specimen to be collected (90.3%) and where to sent notification forms (83.9%) and temperature at which specimen is transported (87.1%). Such low knowledge of a vital surveillance system like AFP surveillance is detrimental to public health and this might be attributed to the poor performance of the surveillance system in Bikita.

Lack of knowledge in the syndromes classified under AFP may have led to low index of suspicion and a lot of cases besides the one picked during records review in the study might have Been be missed/misdiagnosed by the health workers in the district. There was also shortage of AFP notification forms in the district with only two (6.2%0 reporting having the notification forms on the day of data collection. This is contrary to a study in South Africa 2001 by Durrheim et al. were they found out that hospital based nurses were excelling in zero reporting, detection of priority syndromes and prompt appropriate response [6]. Our study findings are also consistent with findings by Ndiaye et al. 2003 in Niger, were they found out that health 50% of workers had two reasons for under reporting namely ignorance of the operations of the system and lack of reporting forms [7] lack of knowledge may be a reason for non detection of AFP cases in Bikita. None of the 31 health workers that we interviewed in Bikita were trained in AFP surveillance and yet are expected to do the surveillance system, this may be another reason for poor performance of the system in Bikita .lack of AFP knowledge was also reported to be a reason for failure to detect cases as shown in Table 4 attached. These finding are also supported by a study by Ndiaye SM et al. on the value of community participation on surveillance, Ndiaye reported that lack of training health workers on surveillance and knowledge were the main challenges to the system [7]. However in a separate study by Varughese P et al. 2005, they found out that knowledge of staff on AFP was high and AFP committees were in place, this reflects the importance of health worker knowledge on surveillance [8].

**Table 4:** Reasons for not detecting AFP cases in Bikita district 2010

**Reason**	**Frequency n = 31**	**%**
Mothers not bringing their children with AFP	10	32.2
No AFP cases occurred in the catchment area	31	100
Not aware of syndromes classified under AFP	19	61

Thirty (96.8%) of the health workers we interviewed in Bikita reported that they felt that both filling notification was their duty and they wanted to continue filling the forms. They however reported need for training and all the 31(100%) mentioned that they need training for them to accurately fill the forms. Willingness of health worker to participate in surveillance is crucial/key in AFP surveillance. This finding is consistent with findings by results of a hospital based study in Shadong, China in which they indicated that improved surveillance requires cooperation of the hospital workers since it is the first port of call for all patients [9].

Only two (6.7%) of the health workers had filled an AFP notification form before and they both felt it was simple and would take less than 20 minutes filling the forms. This contradicts findings by Chimamise et al. 2009 in their AFP surveillance study in Mberengwa district Midlands province where they report that eight health workers who had ever filled an AFP notification form reported filling the forms as time consuming [10]. This might be due to difference in attitudes of health workers. However all health workers felt they needed training and this may affect the running of the system because the health workers feel it is not easy similar findings were also reported by Chimamise et al. 2009 [10].

The participants reported that AFP forms are flexible since they have a space to capture other health problems the AFP case patient may have that are not related to AFP.

None of the study participants were trained in AFP surveillance or even EPI surveillance in Bikita district, The DNO attributes this to the high staff turnover that took place from 2007 to 2009 and some members who were trained left for greener pastures. Another problem that threatened stability of the system was lack of transport and telephones for communication with district office. Majority of health worker use public transport for surveillance purposes 17(54%) of the participants use public transport to sent forms to next level, while 31(100%) reported using telephones but they are using personal cell phones this may prevent them from calling since they do not het airtime. Those who once used public transport to submit T5 forms (forms that summarise all conditions reported at a health facility every month) reported that they were not reimbursed their bus fares and are no longer willing to continue this practice, this will affect the stability of the system even though they are willing to continue with surveillance.

On representativeness we found out that the system was representative since missions, councils and government health centres were participating or involved in surveillance. There are no private clinics or hospital in Bikita

According to the WHO a sensitive AFP surveillance system is one that is able to detect at least 1 case of AFP per 100 000 children under 15 years per year [5,11-13]. Zimbabwe however set a higher standard of two cases per year in the same age group. The system in Bikita is therefore not sensitive similar findings were reported by Chimamise et al. 2009 in AFP surveillance study in Mberengwa were they found out that the system was not sensitive [10].

All study participants reported that the system was useful to the district although they are not holding meetings and use data locally for public health action or continuous improvement (Total Quality Management), they are just gathering data and storing in files and no use is made of the data, this will lead to poor planning of public health activities and actions. No feedback is given to the reporting centres from the district or province. Feedback helps reporting centres know their performance and even other centre’ performances and brings about competition and motivation. The non use of surveillance data for public health action leads to actions that are not evidence based and waste of resources and time.

The cost of running the system is very high given the country’s economy and there is need for partnership in AFP surveillance. The CDC states that in 2003 98 million dollars was provided and 47 million was used for surveillance purposes [12] this shows how crucial surveillance is for polio eradication.

AFP surveillance system in Bikita was acceptable, flexible, not sensitive ad representative, however health worker knowledge on operations of the system was low. However on the other hand the system was not stable, and simple. Data that was collected through the system was not being used to plan public health activities or actions and decision making. The financial resources needed to run the system were huge however it is mainly funded by WHO and UNICEF. Resources to run the surveillance system are not available in the district There was lack of knowledge on syndromes classified under AFP. We therefore recommend;

i) The District Health Executive (DHE) to train all health workers on AFP surveillance and Integrated Disease Surveillance and Response (IDSR).

ii) DMO/PMD to source resources needed for EPI.

iii) DHE to ensure motorized Environmental Health Technician (EHT) assist in reaching hard to reach population.

iv) All should clinics to hold surveillance meetings and use data for public health action at local level.

v) Involve community in AFP surveillance.

## Competing interests

We declare no competing interests.

## Authors’ contributions

KWP designed protocol, collected data, analysed data, wrote report. RF collected data, analysed data, wrote report. MT analysed data, edited report. MM analysed data, developed protocol. All authors read and approved the final manuscript.
